# 

**Published:** 1887-07

**Authors:** 


CONTENTS.

Vol. 34 No. 7.

PAGE
Psycometry....................145
Physiognomy of the Nose........148
Bathing, its Agency in Therapeutics....................150
Epistaxis, or Bleeding of the Nose....................152
Language of the Hand...........154
Physiognomy Illustrated.........155
A Curious Pathological Phenomenon....................156
A New Remedy for Burns.......157
Physical Decay of Cities.........158
The Vassar Girls................159
Spiders.......................159
Wonderful Discoveries...........161
Relation of Exercise to Health...162
Food Adulterations..............163
Red and White..................163
Book Notices...................164
Household.....................165
Miscellaneous..................167

PAGE.
INDEX TO ADVERTISEMENTS OF HALF A PAGE AND OVER.
Winchester's Hypophosphate... I
Hollow Suppositories........... II
Celerina....................... III
Lactopeptine................... IV
Castoria....................... IV
Horsford's Acid Phosphate..... V
Crystalline Phosphate......... VI
Bovinine...................... VII
Goodyear Rubber Co.......... VII
Lactated Food........2d page cover
" " ........3d page cover
New York College of Magnetics.......4th page Cover

